# Editorial: Response to an object near the head/body: Multisensory coding and motor processing guided by sensory systems

**DOI:** 10.3389/fnins.2022.1124062

**Published:** 2023-01-09

**Authors:** Duck O. Kim, Nicholas P. Holmes, Gerome A. Manson, Pavel Zahorik

**Affiliations:** ^1^Department of Neuroscience, University of Connecticut Health Center, Farmington, CT, United States; ^2^School of Psychology, University of Nottingham, Nottingham, United Kingdom; ^3^Sensorimotor Exploration Laboratory, School of Kinesiology and Health Studies, Queen's University, Kingston, ON, Canada; ^4^Department of Otolaryngology and Communicative Disorders, Department of Psychological and Brain Sciences, Heuser Hearing Institute, University of Louisville, Louisville, KY, United States

**Keywords:** sensory coding, stimulus distance, multisensory processing, peripersonal space, dorsal and ventral cortical streams, sensorimotor integration

Detecting and responding to objects near the body in peripersonal space is essential for successful interactions with the environment. Presently, we lack knowledge about the neural coding of the distance of sounds relative to the head, or about the proprioceptive coding of 3-dimensional positions of objects relative to the body. Neural coding of nearby objects is an important subject for neuroscience, and there is a great need to advance knowledge about how sensory and motor systems mediate peripersonal behavior. The present Research Topic drew investigators' attention to the sensory and sensorimotor mechanisms underlying both the perception of objects, and the performance of actions near the body, and to facilitate efforts toward making progress in this area.

We assembled seven original studies and one review article that address various aspects of the Research Topic. This expands the current literature on the sensory and sensorimotor mechanisms underlying behaviors dealing with objects and stimuli near the body.

Effects of active and guided exploration for a sound source on the perceived distance in peripersonal space (0.4–1.5 m from the head; see Figure 1) were investigated by Hug et al. This study is a welcome addition to the present topic areas because it engaged multisensory systems (auditory, tactile, and proprioceptive) and sensorimotor processes. The participants who could actively explore the sound source improved their distance judgements whereas the performance of those whose arms were guided by the experimenter were less accurate than the active group.

**Figure 1:**
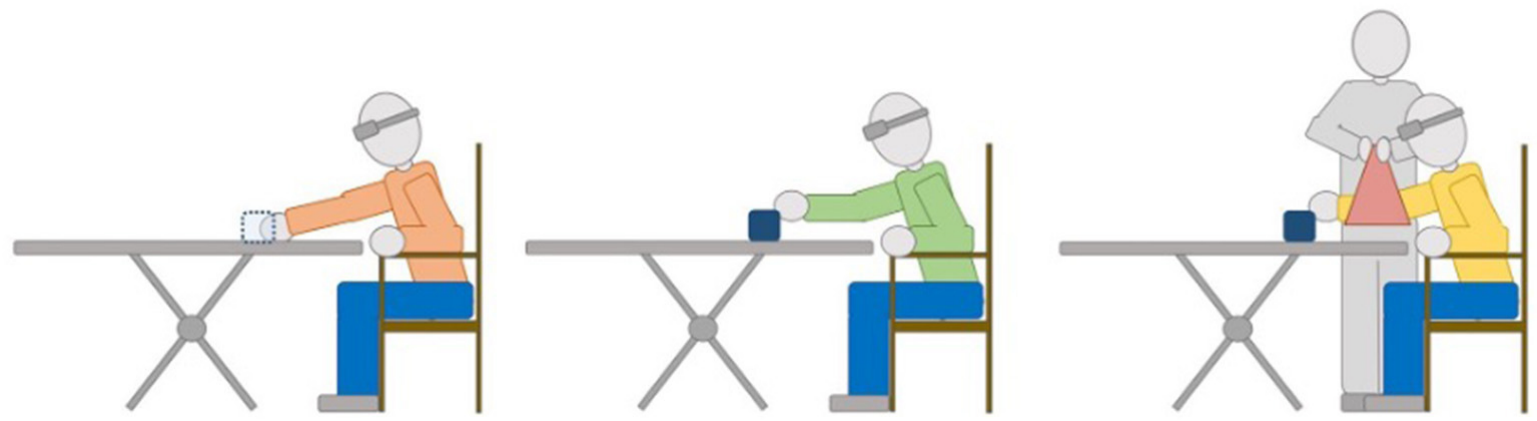
Illustration of the procedure used by Hug et al. Blindfolded participants reached for a nearby sound source. They were divided into three groups that were differentiated by the intermediate training phase: control **(left)**, active **(middle)**, and guided **(right)**.

Zahorik studied audiovisual distance perception in which visual distance “captured” the perceived distance to a sound source. This capture was asymmetric, however. Sound sources farther than the visual target were more frequently judged to be at the distance of the visual target than closer sources. Such results, though fundamentally different than the well-known directional ventriloquist effects, are shown to be predictable by a computational model of auditory-visual distance perception where distance is perceived on a logarithmic scale, and auditory percepts are less precise than visual percepts. These results fill a gap in knowledge in the audiovisual distance perception.

Mooti and Park considered the multisensory factors underlying the perception of head and trunk orientation. In humans, the perception of head-trunk orientation is inherently multisensory (visual, vestibular, and proprioceptive). The relative contributions of the sensory systems in dynamic head-trunk orientation situations are more complicated and less studied than in static situations. The authors addressed the gap in knowledge in the horizontal (yaw) dimension. The results suggest that cervical proprioception is the primary determinant of perceived head-trunk orientation, but that either visual or vestibular information can provide additional information to improve head-trunk orientation accuracy.

Although human reaching behaviors to nearby objects are typically accurate, errors can arise from the initial encoding of the hand or goal location (sensory), the transformation of sensory signals into motor commands (planning), or the movements themselves (execution). Phataraphruk et al. reasoned that initial arm posture would affect execution noise and reach accuracy, and that this would be more prominent when vision was unavailable. Indeed, the size and shape of the distributions of reach responses were determined by more complex interactions involving initial arm posture. Thus, these results provide insight into the multifactorial and multisensory aspects of human reaching behavior.

How the brain integrates information from the eyes and body is the fundamental neuroscience problem tackled by Hsiao et al.. They used virtual-reality methods to manipulate the visual feedback of hand position as people moved. In the study, the real and virtual positions of the hand drifted apart gradually. Participants often made movements according to a combination of the visual and bodily information. Sometimes they became aware of the discrepancy and other times not. They found that small discrepancies were often unnoticed, yet could still affect movement. Larger discrepancies were more often noticed, and participants re-calibrated their movements.

When we place our hand near an object, is our attention allocated automatically to that object? This question is addressed by Reed et al.. Under divided attention, a visual cue presented before a target led to smaller visual-evoked potentials when either the hand or a neutral block was near the target compared to far away. When participants were cued to focus their attention on one side of space, the anchoring effects of the hand or block were not observed. These results raise new questions about how hand location influences visual processing, and about when and where in the brain these effects occur.

Kuroda et al. used a combination of virtual reality and stationary cycling to investigate the visual and proprioceptive contributions to the perception of a passable width during self-motion. The authors replicated previous findings by showing that participants perception of a passable width narrowed as perceived self-motion speed increased. The authors found that optic flow altered judgments of passable width even if participants were not pedaling. The results suggest that visual information about perceived self-motion may contribute more than proprioception to perceptual judgments of passable width.

Lohse et al. reviewed the interactions between the auditory and somatosensory systems, and between the auditory and the motor systems. The review highlights “the importance of considering both multisensory context and movement-related activity in order to understand how the auditory cortex operates during natural behaviors”.

There is a continued need for further research in the areas of multisensory and sensorimotor processes that mediate peripersonal-space behavior. For example, the proprioceptive mechanisms that underlie coding of 3-dimensional coordinates of an object near the body and those of body parts remain to be identified, and how the motor and proprioceptive systems jointly utilize such 3-dimensional information in planning and executing movements of body parts to reach and grasp the target object has yet to be determined. Of additional interest is how information from multiple sensory modalities is combined and weighted to account for differences in the capabilities of each modality. For example, when a salient nearby object is behind, vision is not available. Thus, auditory and vibrotactile detection of such invisible objects would be of vital importance to an organism.

## Author contributions

DK drafted the editorial including summaries of two articles. NH commented on the draft and provided summaries of two articles. GM commented on the draft and provided summaries of one article. PZ commented on the draft and provided summaries of three articles. All authors contributed to the article and approved the submitted version.

